# Sugar Sweetened Beverage Consumption among Primary School Students: Influence of the Schools' Vicinity

**DOI:** 10.1155/2016/1416384

**Published:** 2016-09-26

**Authors:** Alexandre Lebel, Pascale Morin, Éric Robitaille, Benoit Lalonde, Ramona Florina Fratu, Sherri Bisset

**Affiliations:** ^1^Graduate School of Land Management and Regional Planning, Laval University, 2325 Rue des Bibliothèques, Quebec, QC, Canada G1V 0A6; ^2^Evaluation Platform on Obesity Prevention, 2725 Chemin Sainte-Foy, Quebec, QC, Canada G1V 4G5; ^3^University of Sherbrooke, Faculty of Physical Activity Sciences, 2500 Boulevard de l'Université, Sherbrooke, QC, Canada J1K 2R1; ^4^Quebec's National Institute of Public Health, 190 Boulevard Crémazie Est, Montréal, QC, Canada H2P 1E2

## Abstract

The purpose of the research was to explore the associations between the characteristics of schools' vicinity and the risk of sugar sweetened beverage (SSB) consumption in elementary students. Findings exposed an important variation in student's SSB consumption between schools. Schools with a lower socioeconomic status or in a densely built environment tend to have higher proportion of regular SSB drinkers. These characteristics of the school's vicinity partly explained the variation observed between them. We estimated that a student moving to a school with a higher proportion of SSB drinkers may increase his/her chances by 52% of becoming a daily consumer. Important changes in dietary preferences can occur when children are in contact with a new social environment. Findings also support the idea that dietary behaviors among children result from the complex interactions between biological, social, and environmental factors.

## 1. Introduction

Obesity, a level of body fatness associated with risk of developing chronic diseases, such as diabetes, dyslipidemias, and arterial hypertension, has become a serious health concern around the world [1]. Over the last 30 years, the rate of children suffering from obesity has increased significantly. In the United States, 12.4% of preschoolers were estimated to be obese, and this prevalence increased to 20.8% by the age of 14 [2]. In England, the prevalence of obesity was estimated to be 14% for both boys and girls aged 2–15 years [3]. In Canada, the prevalence was estimated to be 11.6% for children in 2010 [4].

Several hypotheses have been proposed for the rapid increase in obesity among children living in occidental countries. On the one hand, fitness levels of children have declined significantly since 1981, regardless of age or gender [5]. At ages 5–11, only 7% of children reach the recommended guidelines of 60 minutes or more of daily physical activity to achieve substantial health benefits [6, 7]. Aside from physical education classes, organized activities such as soccer, swimming, and football as well as active transportation to school and free play should be considered in order to reach daily guidelines [8–10].

On the other hand, overconsumption of energy dense foods with low nutritional quality is seen as an important contributor to childhood obesity [11, 12]. Sugar sweetened beverages (SSB) fall into this category of foods that add calories to the diet but are void of vitamins and minerals [13, 14]. Moreover, since SSBs do not influence appetite, the consumer drinks an additional dose of liquid calories while eating [15]. Convenience and snack food account for 40% of the total calories consumed by US children, of which 22% are from sugar sweetened beverages (SSBs) [16]. In the US, low-income persons consume more sugar drinks in relation to their overall diet than those with higher income [17]. SSB consumption is higher among African-American and Hispanic children and adolescents than among whites [17]. Consumption is higher among US boys than girls; 70% of boys aged 2–19 years consume SSBs daily [18]. A meta-analysis of cohort studies found that higher intake of SSB among children was associated with 55% (95% CI 32–82%) higher risk of being overweight or obese compared with those with lower intake [19]. SSB consumption was also associated with type 2 diabetes where individuals are consuming more than 1-2 servings/day and had a 26% greater risk of developing type 2 diabetes than those consuming less than 1 serving/month [20].

Both theoretical and empirical evidence confirm the important role the environment plays in children's health behaviors in general [21–23] and obesity in particular [24, 25]. While most evidence on obesity related dietary behaviors in youth has focused on the influence of sociocultural and economic factors at the household level [21], more recently studies have shown that built and food environments associate with physical activity, healthful eating, and obesity [22, 26–28]. In particular, access to low nutritional foods and a lack of space for outdoor recreation are related to higher rates of obesity in boys and girls [29, 30]. Multilevel models, inspired from social ecological theory, posit that childhood obesity is influenced by energy intake and expenditure patterns embedded within familial and wider community contexts [31]. For example, associations between obesity and the built environment vary by gender, age, socioeconomic status, and population density, while the relationship between neighbourhood built environment and youth obesity risk is mediated by socioeconomic status (SES) [32]. Schools can indeed be located in an environment that discourages physical activity or promotes reliance on convenience stores and fast food restaurants [5, 33–36].

There is some evidence supporting the existence of a relationship between the characteristics of the environment surrounding schools and students' SSB consumption [37, 38]. A Canadian study found SSB consumption to be lower in schools located in communities with a postsecondary education institution (OR = 0.89; *p* = 0.006 comparing no consumption to one), lower in schools reporting to limit availability of SSB (OR = 0.85; *p* = 0.02), and lower among girls (OR = 0.49; *p* < 0.001) but not significantly different between the school settings (rural, suburban, or rural) [39]. However, substantial heterogeneity in study designs, methods, and measurement tools makes it difficult to draw firm conclusions [37]. Many recent studies have cast doubt on the effect of the environment on child dietary behavior [5, 32, 40–42]. In this respect, many other studies suggest that consumption of SSB in schools is insufficient to predict overall consumption [5, 18, 40, 41, 43] and may be associated with other environmental influences. However, environmental influences of SSB consumption on children, beyond the internal environment of the school, are relatively unknown and need to be explored further to inform public policies and interventions for youth health [12, 40–42, 44]. The purpose of this study was to explore the association between characteristics of the schools' vicinity and the risk of SSB consumption for all primary school students living in a Canadian metropolitan area.

## 2. Materials and Methods

### 2.1. Data-Collection Procedure

The metropolitan area of Sherbrooke is located in the southern part of Quebec close to the USA border. It is the 19th largest urban area in Canada, with a population of approximately 200,000 people in 2011. With the help of the Sherbrooke Regional School Board (SRSB), we undertook an exhaustive survey of 40 public primary schools (children aged 5 to 12 years) in 2007-2008. The main purpose of this survey was to assess the most frequent youth behaviors related to diet and physical activity in order to develop school and community health interventions.

An explanatory letter, a self-administered questionnaire, and a return envelope were sent to the parents of all students registered in a SRSB school. The self-administered questionnaire queried eating behaviors and physical activity. Most of the questionnaire items were inspired from previous reliable surveys [45, 46]. The complete questionnaire was submitted to ten content experts for face validity testing. After minor adjustments, ease of completion and feasibility were assessed with a convenience sample of 40 parents of children at two primary schools. The final version of the questionnaire comprised 42 questions and took approximately 40 minutes to complete. Details about the collection procedure have been published elsewhere [47].

### 2.2. Study Population

All SRSB schools were solicited. In order to obtain a homogeneous sample, smallest schools (<40 students) and specialized schools (e.g., for disabled students) were not part of our study population. Every school participated and the parents of 8612 students completed the self-administered questionnaire, providing a participation rate of 79%.

### 2.3. Participants and Outcome

**Figure 1:**
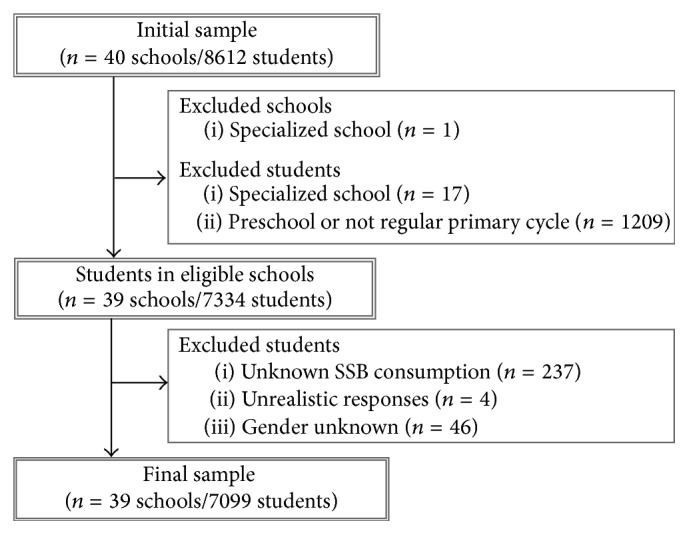
Flowchart of exclusion criteria for schools and students.

Figure 1 presents the detailed eligibility criteria. A total of 7099 students from 37 schools in 39 buildings were kept for the analyses (two schools were actually operating two separate buildings). For our study purpose, each building is kept independent and will be referred to as a school.

#### 2.3.1. Outcome Variable

The outcome variable was a dichotomous indicator of the consumption of at least one sugar sweetened beverage (SSB) per day as compared to less than one SSB per day. Drinking over one SSB per day has been associated with individuals' health status in several recent studies [35, 48, 49]. SSB was measured by consumption of fruit flavored drinks, regular soft drinks, slush, sport drinks, and energy drinks on a 5-point scale ranging from never to several times per day (100% fruit juice was excluded). The individual-level measurement of SSB consumption frequency was adapted from the Canadian Health Measures Survey (CHMS) and the Social and Health Survey on Children and Teenagers (SHSCT). Overall 14.9% reported to drink at least one SSB each day (Table 1).

#### 2.3.2. Individual Covariates

Since SSB consumption is often associated with individual characteristics [39, 50], covariates include gender, academic cycle, cultural origin, and the participation in organized physical activities. No information was available on the household. Table 1 presents the descriptive statistics of the final sample. The sample included the same proportion of girls and boys and approximately a third of students in each of the three academic cycles with slightly more students in the 3rd cycle (10 to 12 years old). The majority of students were Quebec native and francophone (86.5%). Most children were reported to participate in organized physical activities (54.4%).

#### 2.3.3. School-Level Variables

Reported associations between children's dietary behaviors and school neighbourhood measurements presented substantial heterogeneity in the literature [22, 27, 28, 32, 51]. It is important to recall, however, that the contextual influences on child's behaviors are not necessarily direct and causal; it is rather suggested to be considered as a risk regulator. They are not themselves risks but are the conditions that regulate or control exposure probabilities to behaviors that lead to disease [52]. Consequently in this study, the selected contextual variables included not only measurements typically associated with SSB consumption (the access to convenience stores and fast food restaurants), but also other contextual characteristics more broadly associated with children's healthy lifestyle such as a pedestrian friendly environment measured by the presence of green spaces and the walkability of streets [53].

More precisely, six contextual variables measuring characteristics of the built environment surrounding the schools were included in our study: (1) convenience stores density, (2) fast food restaurant density, (3) closest convenience store, (4) closest fast food restaurant, (5) degree of vegetation cover, and (6) street walkability. Data to create these measures were derived from a geographical information system (GIS) using ArcGIS 10 (ESRI Inc., Redlands, CA, USA) software and its Network Analyst extension. These contextual measurements referred to the proximal environment of each school instead of the administrative boundaries of their neighbourhood. In this way, schools measurements were directly comparable. Two global contextual variables were created from these six measures using a principal components procedure. Finally, one school-level variable measuring school socioeconomic index was included in our model.

Density measures of convenience stores and fast food restaurants, degree of vegetation cover, and walkability relied upon the construction of 750 meters of (approximately half a mile) sausage network buffer around the school buildings [54]. Closest access to convenience stores and fast food restaurants was calculated using “Adresses Québec” (http://adressesquebec.gouv.qc.ca/) network file (excluding highway network) and including pedestrian trails from the components of regional geographic use layer. Detailed information on their construction is reported elsewhere [55].

*Convenience Stores Density*. Convenience stores were identified through the Quebec Ministry of Agriculture, Fisheries and Food 2009 sales licenses as well as an automated and manual search by keywords. The convenience store indicator refers to establishments comprised within an area less than 400 square meters, which sell food of all kinds, with or without gasoline sales. The density of convenience stores was computed by estimating the number of stores by km^2^.

*Fast Food Density*. The fast food restaurants indicator combined the “snack,” “takeout restaurants,” and “fast food restaurants” categories, as identified by the Quebec Ministry of Agriculture, Fisheries and Food 2009 sales licenses. These restaurants typically offer limited or no table service and a variety of SSB. The density of fast food restaurants was computed by estimating the number of such restaurants by km^2^.

*Closest Convenience Store*. The distance to the school's closest convenience store was computed on the pedestrian road network.

*Closest Fast Food Restaurant*. The distance to the school's closest fast food restaurant was computed on the pedestrian road network.

*Vegetation Cover*. The normalized difference vegetation index (NDVI) was estimated using a mosaic of 30 meters of spatial resolution images. Images were taken with the Landsat Satellite Enhanced Thematic Mapper Plus (ETM +) sensor in 2011. Cloud presence was minimized by the combination and cutting several scenes. The NDVI values extend from −1 to 1; −1 indicated a total lack of vegetation, while 1 would refer to a dense forest cover. Extraction raster file cell values operations were performed using the Geospatial Modeling Environment software [56].

*Street Walkability*. The walkability index was computed based on four school-level measurements: residential density; destinations density; street connectivity; land-use mix. These indicators were objectively determined for each school [57, 58] (Table 2). Standardized *z*-scores of each measure were summed to construct a walkability index [59, 60]. A higher value of the walkability index suggests a pedestrian friendly environment.

*Global Indexes*. In order to consider schools' neighbourhood built environment measurements more globally, we produced two synthesised contextual indicators issued from a principal component analysis (PCA) [61, 62]. The six built environment variables were introduced in a PCA and produced two uncorrelated factor scores with an eigenvalue higher than 1 (i.e., a significant part of the variance of all six variables was explained by the factor). The strength and the sign of the correlations were used to interpret the meaning of the underlying concept for each factor (Table 3). Interpretation suggested the first factor would be related to the “urban density” or the physical accessibility to food resources and account for about 69% of the variance of the six variables. The other factor refers to the “food sources proximity” and explained 19% of the variance.

*School Socioeconomic Index*. The school socioeconomic index (SSEI) was computed with two variables issued from the 2006 Canadian census by the Quebec Ministry of Education [63]: the education level of the mother and the inactivity of the parents, and it was adjusted for school year 2007-2008 population. These two variables were chosen since they showed the highest correlation with youth academic failure [64]. The SSEI is calculated for all schools of the province and is typically distributed in deciles, from 1 (10% most privileged) to 10 (10% most deprived). According to their provincial score, the 39 schools were reclassified into terciles to distinguish high and low SES among Sherbrooke's schools (Table 1). School-level measurements and factor scores were divided into quartiles to control for the nonnormality of distributions and the nonlinear associations with child SSB consumption.

### 2.4. Data Analysis

Multilevel logistic modeling procedures were used to assess whether variation in SSB consumption could be associated with the neighbourhood's characteristics surrounding the school. The models were fitted using Bayesian estimation procedures as implemented via Monte Carlo Markov Chain (MCMC) methods using Metropolis-Hastings algorithm in MlwiN 2.25 [65]. A four-step sequential modeling strategy was adopted in order to explain the outcome variation between schools (Table 4).

*Model 1* is a two-level null model. This controls for the nonindependence of observations within schools and estimates the mean correlation of students' SSB consumption associated with the school, without taking into account individual or school characteristics. The null model shows if there is a significant variation of SSB consumption between schools.

*Model 2* is a two-level model which includes characteristics of students. This allows estimating the mean correlation of students' SSB consumption associated with the school while controlling for individuals' characteristics. This model determines if the variation of SSB consumption between schools is simply a result of the individual characteristics of students (gender, age, cultural origin, and physical activity). The reference categories were girls, in 1st cycle, from a French-Canadian cultural origin, who participated in organized physical activities. Since the objective of the study was to explore contextual influences on students SSB consumption, Model 2 was used to compare fixed and random parameters in order to observe the effect of contextual measurements while controlling for individual characteristics.

*Model 3* is an extension of Model 2 but includes schools' SSEI. The reference category was the schools in the lowest SSEI tercile (i.e., high SES). This model determines if the schools' SES explains the variation of SSB consumption between schools.

*Model 4* is an extension of Model 2 but includes schools' neighbourhood measurements. At this step, all six school-level built environment measures and the two global indexes were individually tested (8 different models) in order to identify which aspects of the school vicinity may contribute to explain the between-school variation of SSB consumption. This procedure is essential because even if we analysed the entire population of Sherbrooke's primary schools, our second-level distribution includes only 39 observations. Consequently, the variation between second-level observations is limited and does not allow the inclusion of many second-level variables simultaneously. Moreover, second-level measurements based on geographic location often face multicolinearity problem [62, 66]. Nevertheless, analysing them separately provides valuable information on their respective contribution.

All models present the odds ratio (OR) of SSB consumption with its 95% confidence interval (CI) for each characteristic considered (fixed part). The between-school variance structure (random part) was analysed using the median odd ratio (MOR). The MOR was considered significant when the school-level variance was at least 1.96 times higher than its standard error (SE). The MOR can be interpreted as the increased chances a pupil has to drink at least one SSB per day, if this student was moving from a school with a lower chance to a school with a higher chance of a daily SSB consumption. The epidemiologic interpretation of the MOR is more straightforward than other random parameters issued from multilevel modeling since it can be directly compared to a regular OR and thus evaluate if the school-level effect is more or less important than other variables included in the model [67].

We used the deviance information criterion (DIC) to compare the models goodness of fit. The DIC is a measure of data fitting which considers the number of model parameters [68]. A high DIC for the number of parameters suggests a low model performance. A small DIC difference (≤2) between two models shows equivalency while a larger difference indicates a significant improvement [69].

## 3. Results

The null model considered no variable in the fixed part of the model, while the random part shows a significant variation of SSB consumption between schools. It also revealed that students risk of consuming SSB on a daily basis increased by 72% (MOR = 1.72) if they would move from a school which had a lower rate of SSB consumption to one which had a higher rate of SSB consumption.

Model 2 introduced four individual-level indicators. We observed no significant difference between girls and boys. Older students and those that were not QC French had higher risk of consuming SSB. Students that did not participate in organized physical activities had almost twice the risk of drinking SSB daily (OR = 1.96; IC = 1.68–2.28) compared to students involved in organized physical activities. This model shows that a typical student registered in a Sherbrooke primary school who drinks SSB on a daily basis would be 10 to 12 years old, not QC French, and does not participate in organized physical activities. Taking individual characteristics into account reduced the school-level variance which brought down the MOR to 1.62. Thus, individuals characteristics were not uniformly distributed between schools and this would explain 10% of the risk in SSB consumption at the school level. Moreover, considering students' characteristics increased the goodness of fit importantly shown by the DIC difference of 99.33.

Model 3 introduced only one school-level variable, the school socioeconomic index (SSEI); the associations with the individuals' characteristics remained stable. The school's SSEI was significantly associated with students SSB consumption. Schools with the lowest SSEI had an increased risk of 57% to have more students drinking SSB every day. In the random part, considering the SSEI explained an additional 10% of risk shown by the MOR but did not significantly increase the goodness of fit (the model is still good but was not improved).

Prior to building Model 4, we individually introduced the six built environment variables and the two global indexes (8 different models). None of the built environment measures was significantly associated with SSB consumption. For the two global measurements, only the urban density index issued from the PCA was associated with child's SSB consumption. The association not only was significant but also was shown to be relatively strong with an effect similar to the physical activity level of students. However, no gradient was observed between the level of urban density and SSB consumption; that is, students going to a school with the lowest “urban density” environment (few or no fast food restaurants and convenience stores, with a low street walkability, and a high vegetation cover) had significantly a lower risk to drink SSB than students in the other school environments in Sherbrooke (OR = 1.67 to 1.98). This, however, explained less of the between-school variance (MOR = 1.58) than the SSEI (Model 3) but had a slight increase in the model goodness of fit.

## 4. Discussion

We examined associations between the characteristics of the schools' vicinity and the risk of the SSB consumption in a large sample of primary school students. At the individual level, the prevalence of daily SSB consumption among Sherbrooke's school children was 15%, a relatively low level as compared to what was observed in Europe [70] or in the USA [18]. Individual predictors, age, cultural origin, and physical activity level, were all associated with SSB daily consumption. These results are in line with previous research findings showing that individual characteristics are important predictors of SSB consumption, especially age, physical activity, and economic and cultural origin [17]. Unlike other studies [18] we found no association with the gender of students. Together, individual characteristics explained about 10% of the difference of risk of daily SSB consumption between schools.

At the school level, SSB consumption was also found to vary significantly above students' characteristics. The variation in SSB consumption between schools was explained independently by the SSEI and the urban density where it is located. Students in the lowest SSEI were more likely to drink SSB every day than students in schools with the highest SSEI. The schools' socioeconomic status explained an additional 10% in the risk of daily SSB consumption between schools. This suggests that, above individual characteristics and the school's socioeconomic status, a Sherbrooke student moving from a school with a lower rate of SSB consumption to a school with a higher rate would typically increase his/her risk by 52% to daily consume SSB. Although few studies reported SSB consumption between schools, results are in line with the results of Carter and Dubois [71] which suggested that neighbourhood deprivation was more consistently associated with childhood obesity. This finding also recalls that peer behaviors may have an influence on diet among children [72]. Indeed, Freeland-Graves and Nitzke [73] showed that diet preferences and behaviors may change importantly when children are in contact with a new social environment.

Beside the socioeconomic environment of the school, we also explored six contextual characteristics measured within 750 meters of the schools: the number of fast food restaurants, the number of convenience stores, the walkability, the degree of vegetation cover, the distance to the closest fast food restaurant, and the distance to the closest convenience store. Letting individual characteristics constant, none of them were found to have a significant association with SSB consumption. We further explored the combined effect of these measurements and found that students going to schools with the lowest urban density, globally estimated by few or no fast food restaurants and convenience stores in the vicinity, with a low potential of walkability and with an important vegetation cover, may have twice as much as less chances to daily consume SSB than those in other schools (Model 4). As for the socioeconomic environment, this is in line with other studies findings. In a multilevel analysis, Leatherdale et al. [30] found an increased risk of overweight with more fast food retailers around schools. Ogden et al. [17] indicate that a little less than one-half of sugar-drink kilocalories (48%) are consumed away from home. Of these, 43% are purchased in stores, 35.5% in restaurants or fast food establishments, and 1.4% in schools or day-care settings. Moreover, according to van Hulst et al. [74], who realised a study in a metropolitan Canadian city, supermarkets, fast food restaurants, and convenience stores were more accessible around schools than around residences, as shown by shorter walking distances to and higher densities of each type of food establishment in school neighbourhoods. It was shown that attending a school with a higher density of fast food restaurants/convenience stores than supermarket/specialty store gives a greater likelihood of consuming SSB, after adjusting for individual and contextual covariates.

Although we failed to find associations with specific characteristics of the built environment, this finding suggests that SSB consumption may be influenced by a dense urban environment enclosing a set of characteristics which globally offers a more important variety of SSB sources proximal to a school, where the street network is considered walkable and possibly more attractive for pedestrians where the presence of vegetation is important. This finding is particularly important because it suggests that contextual influences are not necessarily causal links on individuals' behaviors but rather act as risk regulators which facilitated or constrained individuals' choices rather than causing them directly [52] and that global contextual measurements are sometime more efficient to capture the individual-environment dynamics for specific context [75]. In the case of Sherbrooke, for example, the lower risk of SSB consumption by students going to a school located in a low urban density environment may be due to a more important proportion of students taking a school bus to travel between home and school and thus is less solicited by the commercial offer of SSB in the school vicinity, a situation that might not be true elsewhere.

These research findings exposed a significant variation in child's SSB consumption between schools and revealed the important role of the socioeconomic and built environment in the surrounding environment. They are supported by a strong methodological setup. Strengths of this study include the high participation rate of households (79%) and primary schools (100%), allowing generalizability of the findings for the studied area and a possible contribution to elaborate public health or urban planning interventions. The use of multilevel analysis controlled for the shared variance of SSB consumption between schools, but also to analyse its importance as compared to individual-level influences. Using global measurements of the built environment allowed detecting the combined influence of some characteristics that were not individually associated with SSB. This finding clearly supports the hypothesis that contextual influences on child's behaviors are not necessarily direct and causal. The individual-environment dynamics are complex to measure; it may be more helpful for research and intervention to consider contextual influences as the conditions that regulate exposure probabilities to behaviors that lead to disease [52]. Although global contextual measurement may be more difficult to interpret, they may be the more efficient way to explain the contextual influences and may help to avoid finding no relationship where one actually exists (type 2 error).

A few limitations need to be kept in mind for the interpretation of the results. The cross-sectional design of this study does not ultimately reveal the direction of the associations between SSB and environmental factors. Also, although questionnaires were anonymous and confidential, data was reported by the parent instead of directly by the child, introducing possible information bias. No validated French-Canadian questionnaire on the frequency of SSB consumption currently exists, but since the measurement we used was adapted from the two important governmental surveys (CHMS and SHSCT), little doubts remain on its reliability [76]. Data pertaining to the characteristics of home environment (composition of household, socioeconomic status) or the distance between home and school was not collected; this information would have helped to better understand the environments to which students are exposed while on their way to school and permitted controlling for confounding effects of parents' socioeconomic status. Finally, while there is a possible interaction between SSEI and the urban density, it was not statistically feasible to integrate both indicators in the statistical model because of multicollinearity. A larger number of schools (e.g., 100 schools or more) would have allowed testing this interaction in a multilevel model, but we were constrained by the actual number of schools in the area (*n* = 39).

## 5. Conclusion

In Canada, approximately 10% of children are currently obese. Given the immediate and long term consequences of childhood obesity, it is of prime importance to better understand the multiple levels of influence that characterize the obesity epidemic, including the social and built environments. As school settings are among the most important environments to which children are exposed to, estimating their impact on dietary choices is essential to understand how the imbalance between energy intake and energy expenditure occurs. Only a few studies have investigated the role of nonresidential environments. To our knowledge, this is the first study to investigate this relationship for an exhaustive set of school at the regional level. Our results revealed important variations of children SSB consumption between Sherbrooke's schools according to the environmental characteristics in its vicinity. These findings are important for health and place studies since they highlight the influence of the school's vicinity taken globally, rather than direct causal links with specific characteristics. Thus it supports theories proposing that dietary behaviors are a result of complex interactions between biological, social, and environmental factors [26]. Since all Sherbrooke's schools participated in the survey, results may be directly used by local and regional stakeholders aiming to plan an intervention regarding schools proximal built environment.

## Figures and Tables

**Table 1:** Characteristics of students and schools areas.

	Frequency
	*n* (%)
*Outcome*	
Sugar sweetened beverages (SSBs)	
At least one SSB each day	1060 (14.9)
Less than one SSB each day	6039 (85.1)

*Students *(*n* = 7099)	
Gender	
Girls	3543 (49.9)
Boys	3556 (50.1)
Primary school cycle	
1st cycle (6-7 years old)	2264 (31.9)
2nd cycle (8-9 years old)	2343 (33.0)
3rd cycle (10-11 years old)	2492 (35.1)
Cultural origin	
Quebec French	6138 (86.5)
Others	717 (10.1)
Unknown	244 (3.4)
Physical activity	
Organized	2309 (32.5)
None	3859 (54.4)
Unknown	931 (13.1)

*Schools *(*n* = 39)	
School socioeconomic index (SSEI)	
High	13 (33.3)
Middle	13 (33.3)
Low	13 (33.3)
Urban density	
Lowest	9 (23.1)
Low	10 (25.6)
High	11 (28.2)
Highest	9 (23.1)

**Table 2:** The walkability index components.

Measure	Definition	Equation	Data source
Residential density	Number of dwellings per hectares	Dwellings number/hectares number of buffer zone	Quebec assessment roll
Density of destinations	Number of destinations per square kilometer	Destinations number/square kilometer of buffer zone	Quebec assessment roll
Density of intersections	Number of intersections per square kilometer	Intersections number/square kilometer of buffer zone	Adresses Québec road network
Land-use mix	Heterogeneity of distribution of square meter footage of commercial, residential, industrial, recreational, and institutional services	Entropy based index	Quebec assessment roll

**Table 3:** Correlation pattern and explained variance of built environment variables by PCA factors.

Built environment variable	Mean (SD)	Correlation pattern		Variance (%)	Underlying concept
		Factor 1	Factor 2		
Nb. fast foods	1.8 (2.3)	**0.894**	−0.093	68.8	Urban density
Nb. convenience stores	1.5 (1.1)	**0.871**	−0.225		
Walkability^†^	0.2 (1.9)	**0.825**	−0.411		
NDVI^††^	0.4 (0.1)	**−0.854**	0.379		

Distance to closest convenience store (m)	1653 (2800)	−0.220	**0.959**	19.1	SSB sources proximity
Distance to closest fast food restaurant (m)	877 (1619)	−0.285	**0.940**		

^†^Walkability index: a higher value of the walkability index suggests a pedestrian friendly environment.

^††^Normalized difference vegetation index: the NDVI values extend from −1 to 1; −1 expressed a total lack of vegetation, while 1 would report a dense forest cover.

**Table 4:** Sequential modeling strategy.

	Null model			Model 2			Model 3			Model 4		
	OR	*95% *CI		OR	*95% *CI		OR	*95% *CI		OR	*95% *CI	
*Fixed part*												

*Students *(*n* = 7099)												
Gender												
Girls				1.00			1.00			1.00		
Boys				0.99	*0.87*	*1.13*	0.99	*0.87*	*1.14*	0.99	*0.87*	*1.13*
School cycle												
1st cycle				1.00			1.00			1.00		
2nd cycle				**1.26**	*1.06*	*1.51*	**1.27**	*1.07*	*1.52*	**1.27**	*1.06*	*1.51*
3rd cycle				**1.46**	*1.23*	*1.73*	**1.48**	*1.24*	*1.76*	**1.47**	*1.24*	*1.74*
Cultural origin												
QC French				1.00			1.00			1.00		
Others				**1.26**	*1.02*	*1.56*	1.23	*0.99*	*1.53*	**1.25**	*1.01*	*1.55*
Unknown				**1.70**	*1.24*	*2.33*	**1.67**	*1.22*	*2.29*	**1.68**	*1.23*	*2.31*
Physical activity												
Organized				1.00			1.00			1.00		
None				**1.96**	*1.68*	*2.28*	**1.95**	*1.68*	*2.26*	**1.95**	*1.68*	*2.26*
Unknown				**1.79**	*1.47*	*2.18*	**1.79**	*1.47*	*2.19*	**1.78**	*1.46*	*2.16*

*Schools *(*n* = 39)												
SES												
High							1.00					
Middle							0.87	*0.59*	*1.28*			
Low							**1.57**	*1.08*	*2.28*			
Urban density												
Lowest										1.00		
Low										**1.98**	*1.19*	*3.29*
High										**1.67**	*1.03*	*2.69*
Highest										**1.88**	*1.16*	*3.06*

*Random part*												

Variance (SE^†^)	0.325 (*0.101*)			0.255 (*0.085*)			0.191 (*0.070*)			0.228 (*0.079*)		
MOR^†^	1.72			1.62			1.52			1.58		
DIC^†^	5796.5			5696.7			5696.4			5694.7		
DIC change	—			99.83			0.275			2.019		

^†^SE: standard error, MOR: median odd ratio, and DIC: deviance information criterion.
